# Pulsed-Field Ablation Using a Novel Ablation-Mapping Integrated System for Pulmonary Vein Isolation—A Preliminary Animal Study

**DOI:** 10.3390/jcdd9120425

**Published:** 2022-11-29

**Authors:** Zhihong Zhao, Yonggang Chen, Bin Wu, Gaodong Qiu, Liangjie Hong, Xinhua Chen, Xingwei Zhang

**Affiliations:** 1 Department of Cardiology, The Affiliated Hospital of Hangzhou Normal University, Hangzhou 310015, China; 2 Key Laboratory of Pulsed Power Translational Medicine of Zhejiang Province, Hangzhou 311121, China; 3 Hepatobiliary and Pancreatic Surgery, The First Affiliated Hospital of Zhejiang University, Hangzhou 310009, China

**Keywords:** pulsed-field ablation, cardiac electrophysiology, pulmonary veins, superior vena cava, arrhythmia

## Abstract

Objective: The purpose of this study is to evaluate the preliminary safety and effect of a pulsed electric field (PEF) ablation system. Methods: The pulmonary veins (PVs) and superior vena cava (SVC) were isolated with the pulsed field ablation (PFA) system, which included a PEF generator and an electrode. The effects of PFA were investigated in six porcines using a novel circular catheter with combined functions (mapping/ablation) designed to work with a cardiac mapping system. The PEF generator delivered a train of biphasic pulsed electric pulses with a high amplitude (800–2000 V) and short pulse duration. The voltage mapping, PVs and SVC potentials, ostial diameters, and phrenic nerve and esophagus viability data were collected 4 weeks later, after which the animals were subsequently euthanized for gross histopathology analysis. Results: PFA 100% isolated the PVs and SVC with four applications with a mean pulse number of 100–150 pulses, causing no muscle convulsion. PFA does not cause PV stenosis or phrenic nerve dysfunction. Histological analysis confirmed 100% transmurally without any venous stenoses or phrenic injuries. Pathology follow-up showed that PFA had selectively ablated cardiomyocytes but spared blood vessels, the esophagus, and phrenic nerves; after ablation, the myocardial tissue showed homogeneous fibrosis. Conclusion: The PFA system is safe and feasible in the preliminary porcine model, which can effectively isolate PVs and SVCs. Transmural tissue damage can be achieved without phrenic palsy or stenosis.

## 1. Introduction

Atrial fibrillation (AF) is defined as a supraventricular tachyarrhythmia with uncoordinated atrial electrical activation and consequently ineffective atrial contraction [1]. The increased incidence of AF is accompanied by stroke, heart failure, and death [2,3,4]. A national survey reported an AF prevalence rate of 1.8% among adults aged ≥45 years in China, and national strategies that focus on the prevention, detection, awareness, and treatment of AF are urgently required nationwide [5]. The AF ablation procedure targets the myocardial sleeve of the pulmonary veins (PVs); AF catheter ablation for PV isolation (PVI) is regarded as the basis for AF ablation to improve symptoms in selected patients with symptomatic [6,7]. As an alternative to radiofrequency ablation, the pulsed electric field (PEF) technique for ablation of arrhythmias AF is expected to be used clinically because of its safety and effectiveness. Pulsed field ablation (PFA) has been reported in studies [8,9,10], thus, an optimized protocol is highly desired. Additionally, dose and effect aspects need to be further investigated, such as how cellular geometry and electrical field orientation significantly affect the real-time electric field distribution, so that the dosage-based mechanism can be fully understood. This approach delivered higher energy electric fields (1600 V/cm) with a pulse width of 30 μs, which can cause transmural non-thermal injury and eliminate collateral gas pulp, muscle convulsions, and thermal injuries observed with microsecond PFA. A novel ablation-mapping integrated catheter can reduce the operation time.

The purpose of this study is to explore a novel PFA system composed of a flexible circular catheter. At the same time, the cardiac three dimensional (3D) mapping electrophysiology (EP) system was also tested for cooperativity. We use our self-developed PFA system to implement electrical isolation of the pulmonary vein and superior vena cava in a porcine model, evaluating its durability after 4 weeks.

## 2. Materials and Methods

### 2.1. Animal Procedure

The experiment was verified by the Technical Committee on Laboratory Animals of the Zhejiang Animal Center (approval number ZJCLA-IACUC-20020024) and followed the Guideline for the Care and Use of Laboratory Animals of Zhejiang University. Six porcine (5 female and 1 male; age, 3.5–5.5 months old; and weight, 55 kg). The subjects underwent baseline left atrium (LA) computed tomography angiography (CTA) imaging (Artis Zee; Siemens Healthineers, Erlangen, Germany) before the ablation procedure to provide images of the PVS anatomy to evaluate the diameter of the PVs ostium and to guide the ablation targets. From the day before and through 3 days after PFA, the porcines were given carprofen (4 mg/kg orally), fentanyl (50 µg/h transdermally), and buprenorphine (0.01 mg/kg intramuscularly) for analgesia. Heparin was administered to maintain the activated clotting time between 250 and 350 s during the procedures. After anesthesia, the porcine was fixed in a regular supine position on the animal operating table and underwent endotracheal intubation, ventilator-assisted mechanical ventilation, and the recording of an ECG (Abbott, Chicago, IL, USA).

### 2.2. PFA System and Catheter

**Figure 1:**
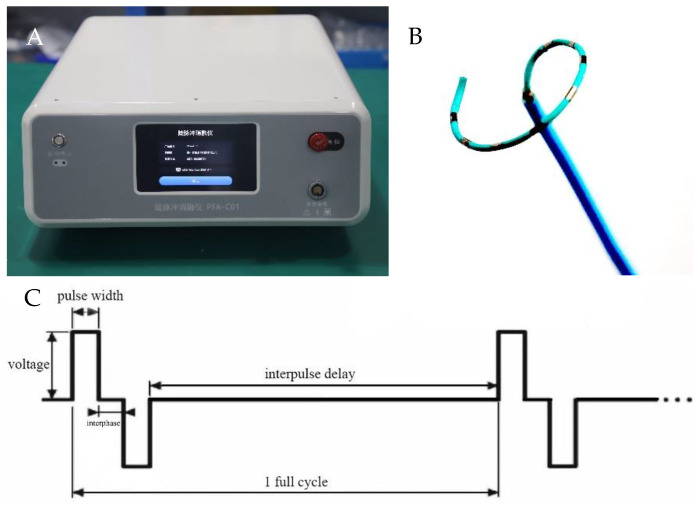
PFA system. (**A**) The biphasic pulse generator was made by Hangzhou Ruidi Biotechnology and weighs 10 kg. (**B**) The mapping/ablation catheter was a 6-French multielectrode flexible circular catheter used for alternating current, bipolar PFA deliveries; the circular catheter includes three models (15, 20, and 25 mm). (**C**) A complete pulse includes positive and negative pulses with the same pulse amplitude and pulse width. The interval between positive and negative pulses is called the interphase. The pulse can be released at a fixed frequency or at each ventricular refractory period.

The PEF ablation system used in this study included a proprietary pulse generator (PFA-C01; Hangzhou Ruidi Biotechnology, Hangzhou, China) to produce electric pulses delivered by a novel flexible circular PFA catheter (PFA-DJ01; Hangzhou Ruidi Biotechnology, Hangzhou, China) (see Figure 1), allowing for the positioning of the catheter over a wide range of PVS ostia sizes. The catheter was adjustable, curving 90°. PEF parameters include pulse voltage 800–2000 V. Each PFA application includes a biphasic-bipolar fashion PEF (1600 V/cm) and PFA waveforms with a pulse width sequence of 50 μs. Pulses were delivered during the ventricular effective refractory period. During the study, the number of electrical pulses output by PEF ablation was 150 to create a sufficient ablated area.

### 2.3. Mapping and Ablation

Percutaneous femoral vein puncture was performed, and two 6-French fixed-curve sheaths (St. Jude Medical, St. Paul, MN, USA) were placed in the left and right femoral veins, respectively, and the reference electrode was pasted on the body surface outside the thorax as a reference for EnSite Precision EP system positioning. Using a trans-septal needle (BRK; St. Jude Medical, St. Paul, MN, USA) through the SL1 sheath and under digital subtraction angiography (DSA) guidance, the trans-septal puncture was performed and a bi-directional guiding sheath (Carto Vizgo^®^, Biosense Webster, Wilmington, Delaware, USA) was exchanged into the LA, which is essential for localization and ablation of the PVs, including the right superior PVs (RSPV) and the common inferior pulmonary vein (CIPV). RSPV and CIPV ostium, superior vena cava (SVC), anatomical, and voltage mapping were conducted with the PFA catheter to evaluate electrograms before and after each PFA application to assess for electric isolation. PFA applications were delivered with voltage amplitudes of 800–1000 V. The PVs, including RSPV, CIPV, and SCV, were selected in each of the six porcines to receive PFA. To ensure accurate and consistent positioning, all catheter placements were guided by DSA and EnSite 3D mapping with magnetic positioning function. Across the six subjects, the intracardiac multi-conductance electrocardiogram was recorded, and the criteria for effective PFA were met, including the electrogram amplitude being reduced to <0.5 mV and the applications with electrogram amplitude loss >50% being included in the statistical analysis. The data were collected during ablation, including electrograms, and intracardiac electrograms. The generator delivered pulses to proximal PVs and an SVC site during the ventricular refractory period. To verify the effects of PFA, the exit block was tested by PFA catheter pacing, and voltage mapping before and after ablation was used to verify the PFA effects too [11]. The percentage of electrogram amplitude reduction after ablation was calculated and compared to the pre-ablation value [12].

### 2.4. Gross Pathology and Pathological Analysis

Four weeks after ablation, the ablated myocardial tissue, adjacent esophagus, and phrenic nerve were submitted for analysis. Tissues were trimmed, and pertinent sections were fixed in 10% formalin, and PVs, SVCs, esophagus, and phrenic nerve sections were obtained. Slides were stained with hematoxylin and eosin and Masson’s trichrome staining. The evaluation of local healing responses was included in the histopathologic assessment. The pathological changes in PVs and SVC sections, edema, and thrombus were evaluated under a microscope [13].

### 2.5. Statistical Analysis

Continuous variables are expressed as mean ± SD or median with interquartile range (IQR), while categorical variables are represented by count and percentage. Continuous variables were compared between the groups *via* the Mann–Whitney U test or Student’s t-test, and categorical variables were compared by chi-square analysis or Fisher’s exact test, as appropriate. A *p* < 0.05 was considered significant. Statistical analyses were performed with IBM SPSS Statistics Version 20.0 (SPSS Inc., Chicago, IL, USA).

## 3. Results

### 3.1. PFA Applications and Dosages

In this study, six subjects were included and 2–4 trains of PFA application were delivered with a median dosage for each application of 150 (range, 100–250) pulses. Six SVC and 12 PVs were ablated, and a total of 66 PFA applications were delivered to the RSPV, CIPV, and SVC; the first porcine received two applications/site, and the subsequent five porcines received four applications/site. All applications were successfully delivered, and none resulted in waveform discontinuities indicative of arcing. Importantly, sustained porcine atrial arrhythmias were observed (Table 1). 

### 3.2. Electrogram, Voltage Mapping, and Complications

**Figure 2:**
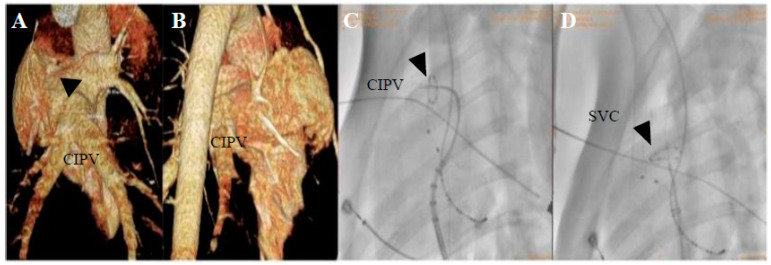
Pulmonary vein computed tomography angiography (CTA) and DSA images with PFA are shown. (**A**,**B**) CTA imaging for the CIPV anatomical measurements. (**C**,**D**) DSA is used for catheter positioning, CIPV and SVC ablations were performed during PFA.

**Figure 3:**
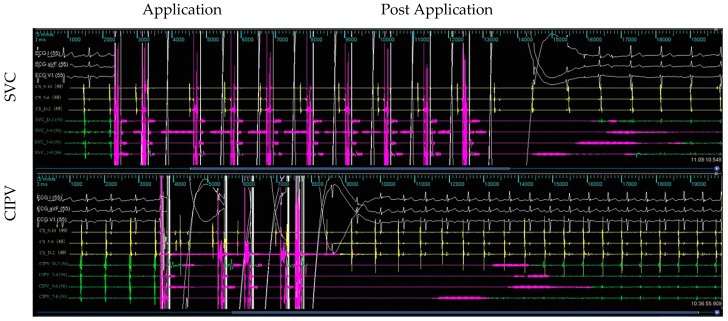
Acute isolation of the SVC and CIPV. PFA is recorded from bipolar electrograms from the 10-electrode array; the PFA catheter is placed in the ostial of the SVC and CIPV. Electrogram signal reduction in the SVC and CIPV with PFA across the CIPV and SVC ostial treatment targets, only far-field signals remained. Complete electrical isolation of all structures was confirmed by the entrance block. White color for ECGI, avF, V_1_ leads; yellow color for coronary vein sinus electrode potential; green color for PFA catheter electrode potential.

**Figure 4:**
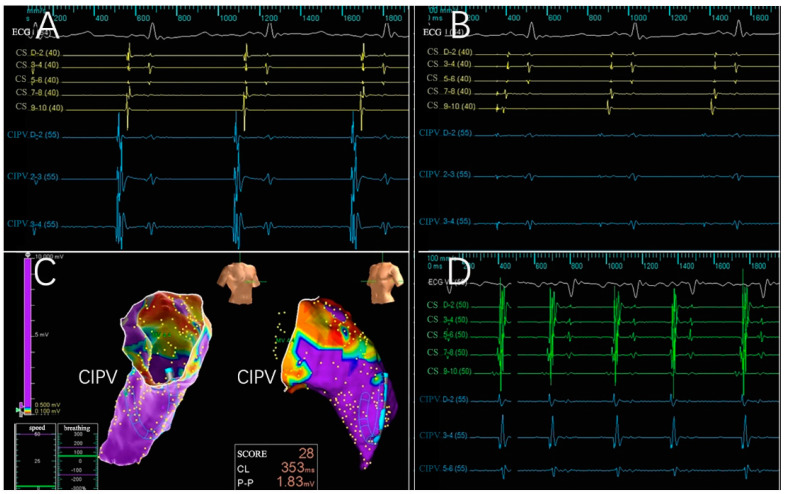
Electrophysiologic evaluation of the CIPV treatment with PFA. Electrograms for the CIPV were reduced, and after trains were delivered, only far-field signals remained. (**A**,**B**) Electrograms before and after PFA; complete electrical isolation was confirmed by the entrance block. (**C**) A chronic map demonstrating isolated CIPV. (**D**) A potential reduction was observed 4 weeks after PFA, showing a level of isolation in the CIPV. ECG:Electrocardiogram; CS: coronary vein sinus; CIPV: common inferior pulmonary vein.

**Figure 5:**
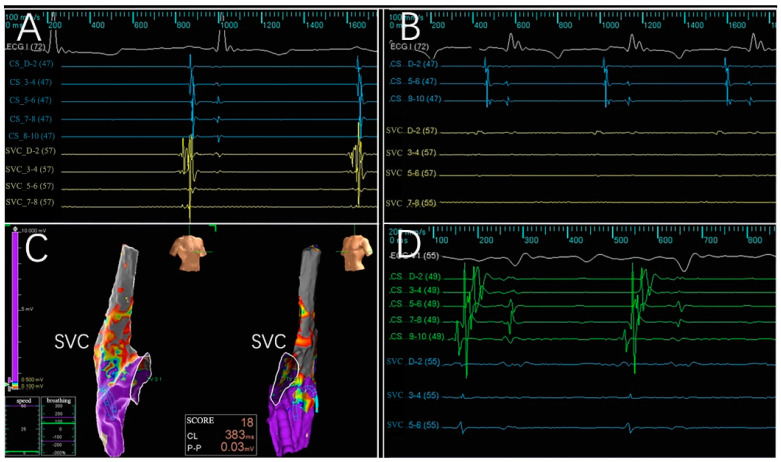
Electrophysiologic evaluation of the SVC treatment with PFA is shown. Electrograms for the SVC were reduced after the trains were delivered. (**A**,**B**) Electrograms before and after PFA; complete electrical isolation was confirmed by the entrance block. (**C**) A chronic map demonstrating isolated SVC. (**D**) Electrograms 4 weeks after PFA. ECG:electrocardiogram; CS: coronary vein sinus; SVC: superior vena cava.

Across the six subjects, 66 intracardiac multi-conductance electrocardiograms were recorded, and the applications with electrogram amplitude loss >50% totaled 60 (100.0%) and were included in the statistical analysis (Table 1). During the ablation process, no hemodynamic abnormalities occurred, and only one atrial arrhythmia occurred. Furthermore, the PFA catheter was relocated in the PVs and SVC 4 weeks after ablation; no atrial electrical activity was recorded, indicating that atrial electrical activity cannot be conducted to the PVs and SVC (Figure 2, Figure 3, Figure 4 and Figure 5).

### 3.3. Gross Pathology

**Figure 6:**
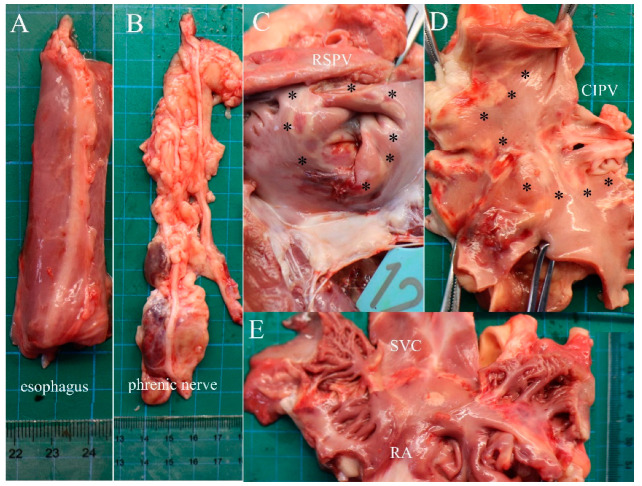
Pathology. (**A**) Outer esophagus. (**B**) The outer phrenic nerve. (**C**–**E**) Internal aspect of the transmural lesions in the RSPV, CIPV, and SVC. Asterisks indicate the ablation area.

**Figure 7:**
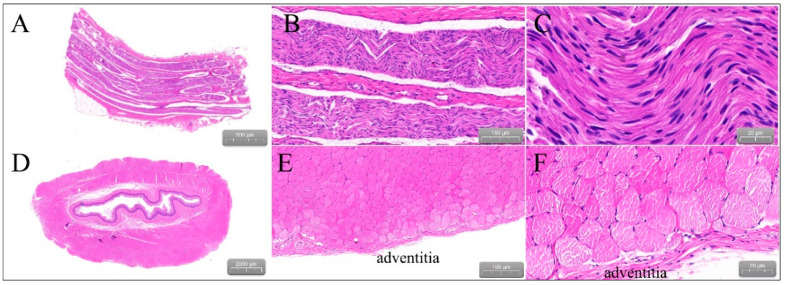
Hematoxylin and Eosin (E&A) staining. (**A**–**C**) Represent the phrenic nerve. (**D**–**F**) Represent the esophagus. Note that the cell boundaries are clear and neatly arranged without any damage.

**Figure 8:**
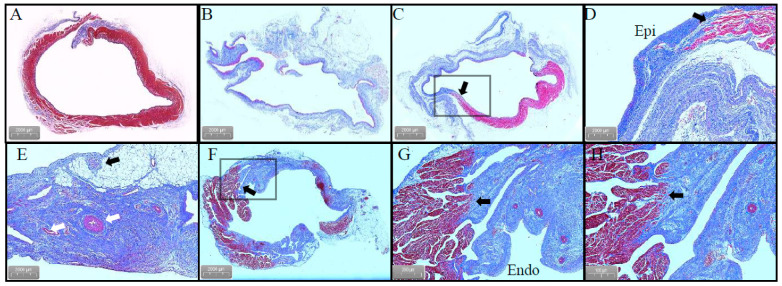
Masson’s trichrome stain. (**A**) Normal PVs tissue. (**B**) Transmural damage to the RSPV tissue caused by PFA without stenosis. (**C**,**D**) The arrows point to transmural cardiomyocyte loss and an area with a relatively unaffected myocardium. (**E**) White: vascular structure; black: nerve tissue. (**F**) Transmural damage to the SVC caused by PFA. (**G**,**H**) The arrows point to transmural cardiomyocyte loss and an area with a relatively unaffected myocardium. Abbreviations: Epi, epicardial; Endo, endocardial.

**Figure 9:**
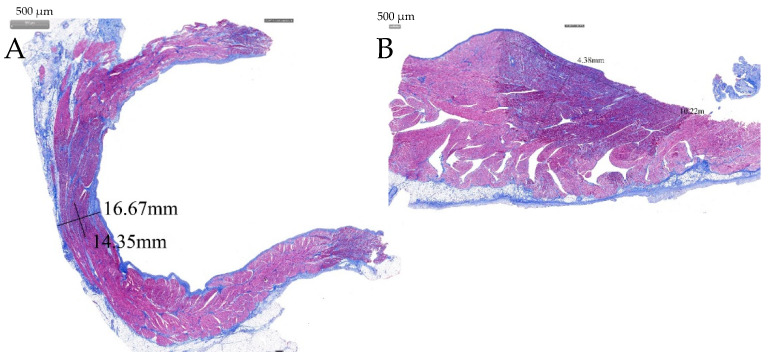
Masson’s trichrome stain. (**A**) In a longitudinal section along the RSPV lesions, a transmural lesion is seen in the RSPV. (**B**) Section demonstrating non-transmural lesions in the SVC.

The ablated porcines were dissected to check the gross pathological status of the PVs and SVC in the ablation area; the esophagus and phrenic nerve were observed too (Figure 6). The esophagus and phrenic nerve were not affected. Considering the PVs and SVC, the intimal surface was smooth and intact. No PVs or SVC stenosis was found. HE staining demonstrated the phrenic nerve and esophagus cell boundaries were clear and neatly arranged without any damage (Figure 7). The ablation area was stained unevenly with fibrous tissue. The blood vessels and nerves remain intact (Figure 8). The transmurally rates of PVs ranged from 91 to 100% as the target anatomic region ablated from two to four applications/site, and the transmurally rates of SVC were 90%, where both lesion width borders were discernable; the width measurements were 4.38 ± 2.61 mm and 10.22 ± 3.38 mm, respectively (Figure 9).

## 4. Discussion

### 4.1. Features of PFA Systems

This study involved a combination of doctors and an engineering multi-disciplinary innovation team. Herein, a novel PEF ablation device was developed with the following innovation points: (1) Our laboratory-independent research and development of chip semiconductors realized a high-voltage, ultrashort pulse generator with independent intellectual property rights; (2) the electrical parameters differ from the existing PFA system, i.e., the PEF is sharper and more energetic; (3) the system adopted an ECG R-wave-triggered synchronous control circuit, effectively reducing the risk of atrial arrhythmias induced during ablation procedures; (4) an adaptive bendable ablation catheter of polymer materials was developed, achieving insulation safety and a convenient operation; and (5) the energy is of a high, ultra-short, and steep pulse. The pulse width of the sharpened square-wave pulse, with the above improvement, allows the current convulsions and air pop caused by microsecond pulses to be effectively avoided. Our PFA system, in the PFA mode, is similar to the non-thermal irreversible electroporation clinically used for tumor treatment, where 50–100 electric pulses of between 10 and 100 μs are delivered at a frequency of 1–2 Hz, contrasting with commercial clinical electroporation devices that deliver voltages of ≤3 kV [14]. As such, pulse parameters (pulse voltage, pulse width, pulse number, etc.) are commonly used to adjust the electric field distribution around electroporation electrodes to generate a larger lesion and enhance the certainty of ablation. Our PFA system’s integration with the mapping system allows the positioning and discharge of the PFA catheter, removing the need for X-rays. The 10-electrode thinner circular mapping/ablation catheter directed by a bi-directional guiding sheath, which can more easily reach the ablation target, may have improved the efficiency of PVI significantly, and there is no need to exchange for a long sheath or repeat an atrial septal puncture; thus, it can greatly shorten the ablation time, i.e., an electrophysiological operation that originally took 2–3 h was shortened to 0.5–1 h [15].

### 4.2. Ablation Effectiveness of the PFA System

In this pre-clinical study, we examined the safety and efficacy of a flexible circular mapping and ablating catheter (PFA-DJ01). Our PFA system is based on magnetic positioning navigation and integrates 3D modeling, mapping, and ablation. With the help of an adjustable sheath, the unique electrodes can easily complete the PVI. In this study, the feasibility of PVI and SVC isolation (SVCI) by PFA with a novel 6-French, flexible, circular PFA catheter was proven. The amplitude and stimulation threshold of the local PVs and SVC potentials were measured by PFA catheter mapping at multiple locations of the PVs and SVC before and after ablation and again 4 weeks after ablation. The PVs and SVC were then sliced, stained, and histologically examined. The results showed that the immediate potential amplitude of PVs and SVC was significantly reduced after PFA; further, the potential reduction was observed at 4 weeks of follow-up. Histological results showed that most of the ablation sites could achieve transmural injury, the inflammation is slight, no stenosis was found in the PVs or SVC, and no significant complications, such as sinoatrial node injury, phrenic nerve injury, or injury to the esophagus, were observed in the immediate and follow-up periods after PFA of the PVs and SVC. Additionally, our previous study showed that the ablation of the myocardial cell death mechanism, myocardial fibrosis evolution, and influence of the ablation area provided an experimental basis for the clinical application of PEF ablation [16]. Relevant studies have shown that in bipolar PFA burst deliveries, the effect of bubbles is negligible [17,18]. PFA has tissue selectivity with non-thermal energy [19].

As a new technology in the field of AF ablation, PFA has three outstanding advantages. First, it is fast; in this study, our system created continuous PVI and SVCI, and the catheter size remained stable, providing predictable and irreversible ablation, with a voltage of ≤0.1 mV predicting irreversible damage. The real discharge ablation time only takes 10 s; it has improved the efficiency by ≥5–10 times; and the operation was completed under sedative anesthesia. The overall operation time is 30 min. Second, the PFA procedure does not affect the esophageal or phrenic nerves. Third, during PFA applications, the ablation area is continuous, independent of the contact force, so the risk of a heart rupture is relatively low. This catheter can perform ablation as well as mapping, and PVI can be accomplished with a single trans-septal puncture. It is time- and cost-effective. This study used domestic innovative medical equipment as well as the system standard series of pre-clinical animal experiments, observed in situ real-time pathological changes, and gathered continuous follow-up in different postoperative periods.

### 4.3. Limitations

The study presents several limitations: (1) The complex intracardiac environment and local dynamic electrical characteristics of tissues will have a certain impact on the PFA zones, so the voltage amplitude and the number of pulses should be adjusted during the ablation process in order to achieve a high degree of durable pulmonary vein isolation; (2) we did not show the early time points, such as 1–3 days after PFA, and the esophageal damage in this article; and (3) the new device needs more evaluation and evidence.

## 5. Conclusions

Our PEF ablation system is safe and feasible in a preliminary porcine model, which can effectively isolate PVs and SVCs. Transmural tissue damage can be achieved without phrenic palsy or stenosis.

## Figures and Tables

**Table 1:** PFA applications and lesion characteristics.

Animal	Rationale for Number of Active Electrodes	Target Anatomic Region and Frequency of Ablation			Electrogram Amplitude Reduction > 50%	Atrial Arrhythmias
		RSPV (n = 22)	CIPV (n = 22)	SVC (n = 22)		
#1	6-8, Circumferential	2	2	2	83.3%	0
#2	6-8, Circumferential	4	4	4	100%	0
#3	6-8, Circumferential	4	4	4	100%	1
#4	6-8, Circumferential	4	4	4	100%	0
#5	6-8, Circumferential	4	4	4	100%	0
#6	6-8, Circumferential	4	4	4	100%	0

## Data Availability

The raw data supporting the conclusions of this article will be made available by the authors without undue reservation.
